# Predicting residue-wise contact orders in proteins by support vector regression

**DOI:** 10.1186/1471-2105-7-425

**Published:** 2006-10-03

**Authors:** Jiangning Song, Kevin Burrage

**Affiliations:** 1 Advanced Computational Modelling Centre, The University of Queensland, Brisbane Qld 4072, Australia

## Abstract

**Background:**

The residue-wise contact order (RWCO) describes the sequence separations between the residues of interest and its contacting residues in a protein sequence. It is a new kind of one-dimensional protein structure that represents the extent of long-range contacts and is considered as a generalization of contact order. Together with secondary structure, accessible surface area, the B factor, and contact number, RWCO provides comprehensive and indispensable important information to reconstructing the protein three-dimensional structure from a set of one-dimensional structural properties. Accurately predicting RWCO values could have many important applications in protein three-dimensional structure prediction and protein folding rate prediction, and give deep insights into protein sequence-structure relationships.

**Results:**

We developed a novel approach to predict residue-wise contact order values in proteins based on support vector regression (SVR), starting from primary amino acid sequences. We explored seven different sequence encoding schemes to examine their effects on the prediction performance, including local sequence in the form of PSI-BLAST profiles, local sequence plus amino acid composition, local sequence plus molecular weight, local sequence plus secondary structure predicted by PSIPRED, local sequence plus molecular weight and amino acid composition, local sequence plus molecular weight and predicted secondary structure, and local sequence plus molecular weight, amino acid composition and predicted secondary structure. When using local sequences with multiple sequence alignments in the form of PSI-BLAST profiles, we could predict the RWCO distribution with a Pearson correlation coefficient (CC) between the predicted and observed RWCO values of 0.55, and root mean square error (RMSE) of 0.82, based on a well-defined dataset with 680 protein sequences. Moreover, by incorporating global features such as molecular weight and amino acid composition we could further improve the prediction performance with the CC to 0.57 and an RMSE of 0.79. In addition, combining the predicted secondary structure by PSIPRED was found to significantly improve the prediction performance and could yield the best prediction accuracy with a CC of 0.60 and RMSE of 0.78, which provided at least comparable performance compared with the other existing methods.

**Conclusion:**

The SVR method shows a prediction performance competitive with or at least comparable to the previously developed linear regression-based methods for predicting RWCO values. In contrast to support vector classification (SVC), SVR is very good at estimating the raw value profiles of the samples. The successful application of the SVR approach in this study reinforces the fact that support vector regression is a powerful tool in extracting the protein sequence-structure relationship and in estimating the protein structural profiles from amino acid sequences.

## Background

A major challenge in structural bioinformatics is the prediction of protein structure and function from primary amino acid sequences. This problem is becoming more pressing now as the protein sequence-structure gap is widening rapidly as a result of the completion of large-scale genome sequencing projects [1,2]. As an intermediate but useful step, predicting a number of key properties of proteins including secondary structure, solvent accessibility, contact numbers and contact order is a possible and promising strategy, which simplifies the prediction task by projecting the three-dimensional structures onto one dimension, i.e. strings of residue-wise structural assignments [3-6].

However, the current state-of-art methods can only achieve a prediction accuracy of 76%-80%, for the three-state secondary structure prediction [7]. One of the main reasons for the limitation to accurate secondary structure prediction is attributed to the long-range residue contacts (described by residue contact order), which is often overlooked or under-represented in the current prediction methods. Kihara examined the relationship between residue contact order and the prediction accuracy and found that there exists a negative correlation for the α-helices and β-strands [8]. Their studies indicated that long-range residue contacts have significant effects on the secondary structure prediction. Therefore, it is worthwhile incorporating these two-dimensional contact maps of residue contact orders in order to further improve the prediction performance. Moreover, in addition to its significance to secondary structure prediction, residue contact order also has an important implication in protein folding rate prediction [9,10]. Previous studies have well established that residue contact order has a strong correlation with folding rate and, more recently, Punta and Rost demonstrated that the two-state folding rates of a protein can be reliably estimated by predicting its residue-residue contacts even for the proteins of unknown structures [10].

Residue-wise contact order (RWCO) is a new kind of one-dimensional protein structure representing the extent of long-range contacts, which is a sum of sequence separations between the given residue and all the other contacting residues [11,12]. Relative contact order (CO) was originally put forward by Plaxco *et al*. to describe the complexity of protein topology and is often used to study the correlation between protein topology and folding rate [13]. Based on this definition, Kihara further defined the residue contact order (RCO), which was the average contact order of the residue of interest [8]. Recently, Kinjo *et al*. put forward a similar definition and introduced the concept of RWCO [11,12], which can be considered as a generalization of RCO. In other words, RWCO is the sum of the sequence separation of contacting residues, that is, for residue *i*, RWCO_*i *_= n × RCO_*i*_, where n is the number of contacting residues with residue *i *[8]. As discussed by Kinjo *et al*., CO is a per-protein quantity based on the whole protein level, while RWCO and RCO are per-residue properties based on the residue level. Recent studies have indicated that it is applicable to use RWCO, together with contact numbers and secondary structures to accurately recover the three-dimensional structures of a protein [6,12]. Therefore, accurate prediction of RWCO values in proteins would have many important applications, especially in protein structure prediction and protein folding rate prediction, as well as helping to determine protein homologous folds.

Several methods have been developed so far to predict the RWCO distributions from the primary amino acid sequences. Kinjo *et al*. proposed a simple linear regression method to predict RWCO values and the local sequence information with multiple sequence alignments in the form of PSI-BLAST profiles was extracted using a sliding window scheme centered on the target residue. Their method achieved a highest correlation coefficient (CC) of 0.59 between the native (observed) and predicted RWCO values using an unusual half window size of 26 (full window size = 53). And the corresponding root mean square error (RSME) was 1.03. This result was averaged on the test datasets by 15-fold cross-validation. They claimed that this long-range correlation reflected by the unusually long window size was a conspicuous property of RWCO, which was distinctly different from any other one-dimensional structure prediction [11]. Later they developed another method called critical random network (CRN) to refine this task using the same extra-large window size of 53 residues, and their accuracy was further improved to a CC of 0.60 and RMSE of 0.88 [12].

In the present study, we proposed a novel method to predict the RWCO profiles from amino acid sequences based on support vector regression (SVR). Different from the linear regression approach, our method uses the non-linear radial basis kernel function (RBF) to approximate and determine the sequence-RWCO relationship. We extensively explored seven different sequence encoding schemes and examined their different effects on the prediction performance. The results showed that introducing the predicted secondary structure by PSIPRED program, in conjunction with the global information such as protein molecular weight and amino acid compositions, could significantly enhance the prediction performance. Our method could predict RWCO values with a Pearson's correlation coefficient (CC) of 0.60 and root mean square error (RMSE) of 0.78. We compared our prediction accuracy with that of Kinjo *et al*. using the same 15-fold cross-validation based on the same training and testing datasets. Our results show that our approach is superior to the linear regression method and slightly better than the critical random network method in predicting protein structural profile values and describing sequence-structure relationships.

## Results

### RWCO distribution at four different radius thresholds

**Figure 1:**
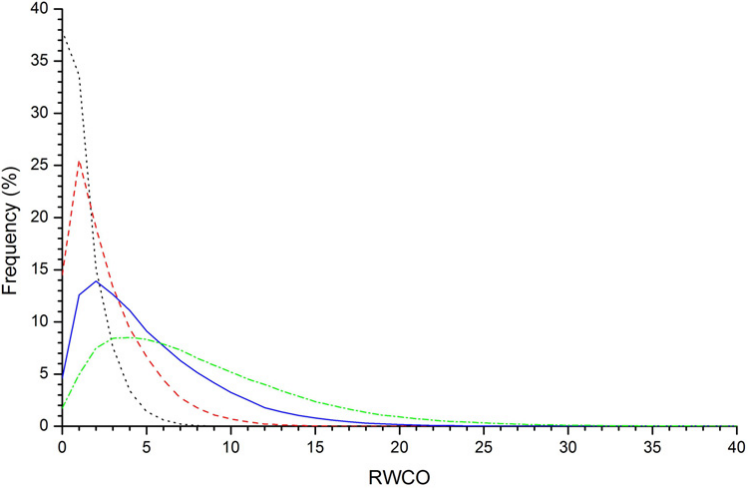
**RWCO distributions at four different radius thresholds**. The radius *r*_*d *_cutoffs are selected as 8 Å, 10 Å, 12 Å and 14 Å, which are represented by dotted black, dashed red, solid blue and dot-and-dashed green lines, respectively.

The RWCO value for each residue in the dataset was computed by defining four different sphere radii *r*_*d *_centered on the *C_β _*atom of the target residue, i.e. *r*_*d *_= 8Å, 10Å, 12Å and 14Å. For each given radius cutoff *r*_*d*_, we computed the average RWCO distributions over the whole dataset using formula (1) and (2), which are displayed in Figure 1. The corresponding mean (N¯
 MathType@MTEF@5@5@+=feaafiart1ev1aaatCvAUfKttLearuWrP9MDH5MBPbIqV92AaeXatLxBI9gBaebbnrfifHhDYfgasaacH8akY=wiFfYdH8Gipec8Eeeu0xXdbba9frFj0=OqFfea0dXdd9vqai=hGuQ8kuc9pgc9s8qqaq=dirpe0xb9q8qiLsFr0=vr0=vr0dc8meaabaqaciaacaGaaeqabaqabeGadaaakeaacuWGobGtgaqeaaaa@2DE9@) and standard deviation (SD) are listed in Table 1. There are significant correlations between the four different RWCO distributions. The RWCO values defined by four different radii cutoffs have correlation coefficients (CCs) all greater than 0.853 (Table 2). It can be seen that RWCO distributions with large radius cutoffs (*r*_*d *_= 12Å and 14Å) are close to gamma distributions (Figure 1) and even after the normalization step using equation (3), their normalized RWCO distribution profiles retain the same tendency. Since previous studies also indicated that larger radii *r*_*d *_= 12Å and 14Å have more significant meaning in protein fold recognition [20] and because the directly related work [11,12] also used a large radius cutoff of 12Å, we set *r*_*d *_= 12Å in the following analysis in order to be consistent with the previous work and make an objective comparison.

**Table 1:** The Mean (N¯
 MathType@MTEF@5@5@+=feaafiart1ev1aaatCvAUfKttLearuWrP9MDH5MBPbIqV92AaeXatLxBI9gBaebbnrfifHhDYfgasaacH8akY=wiFfYdH8Gipec8Eeeu0xXdbba9frFj0=OqFfea0dXdd9vqai=hGuQ8kuc9pgc9s8qqaq=dirpe0xb9q8qiLsFr0=vr0=vr0dc8meaabaqaciaacaGaaeqabaqabeGadaaakeaacuWGobGtgaqeaaaa@2DE9@) and Standard Deviation (SD) of RWCO values according to different radius (*r*_*d*_) cutoffs.

	***r***_***d ***_= 8 Å	***r***_***d ***_= 10 Å	***r***_***d ***_= 12 Å	***r***_***d ***_= 14 Å
N¯ MathType@MTEF@5@5@+=feaafiart1ev1aaatCvAUfKttLearuWrP9MDH5MBPbIqV92AaeXatLxBI9gBaebbnrfifHhDYfgasaacH8akY=wiFfYdH8Gipec8Eeeu0xXdbba9frFj0=OqFfea0dXdd9vqai=hGuQ8kuc9pgc9s8qqaq=dirpe0xb9q8qiLsFr0=vr0=vr0dc8meaabaqaciaacaGaaeqabaqabeGadaaakeaacuWGobGtgaqeaaaa@2DE9@	0.76	2.15	4.51	7.57
SD	1.27	2.35	3.92	5.71

**Table 2:** The correlation coefficients between the different radius (*r*_*d*_) cutoffs.

	***r***_***d ***_= 8 Å	***r***_***d ***_= 10 Å	***r***_***d ***_= 12 Å	***r***_***d ***_= 14 Å
***r***_***d ***_= 8 Å	1.0	0.952	0.912	0.854
***r***_***d ***_= 10 Å		1.0	0.971	0.935
***r***_***d ***_= 12 Å			1.0	0.979
***r***_***d ***_= 14 Å				1.0

### Relationship between accessible surface area and RWCO

**Figure 2:**
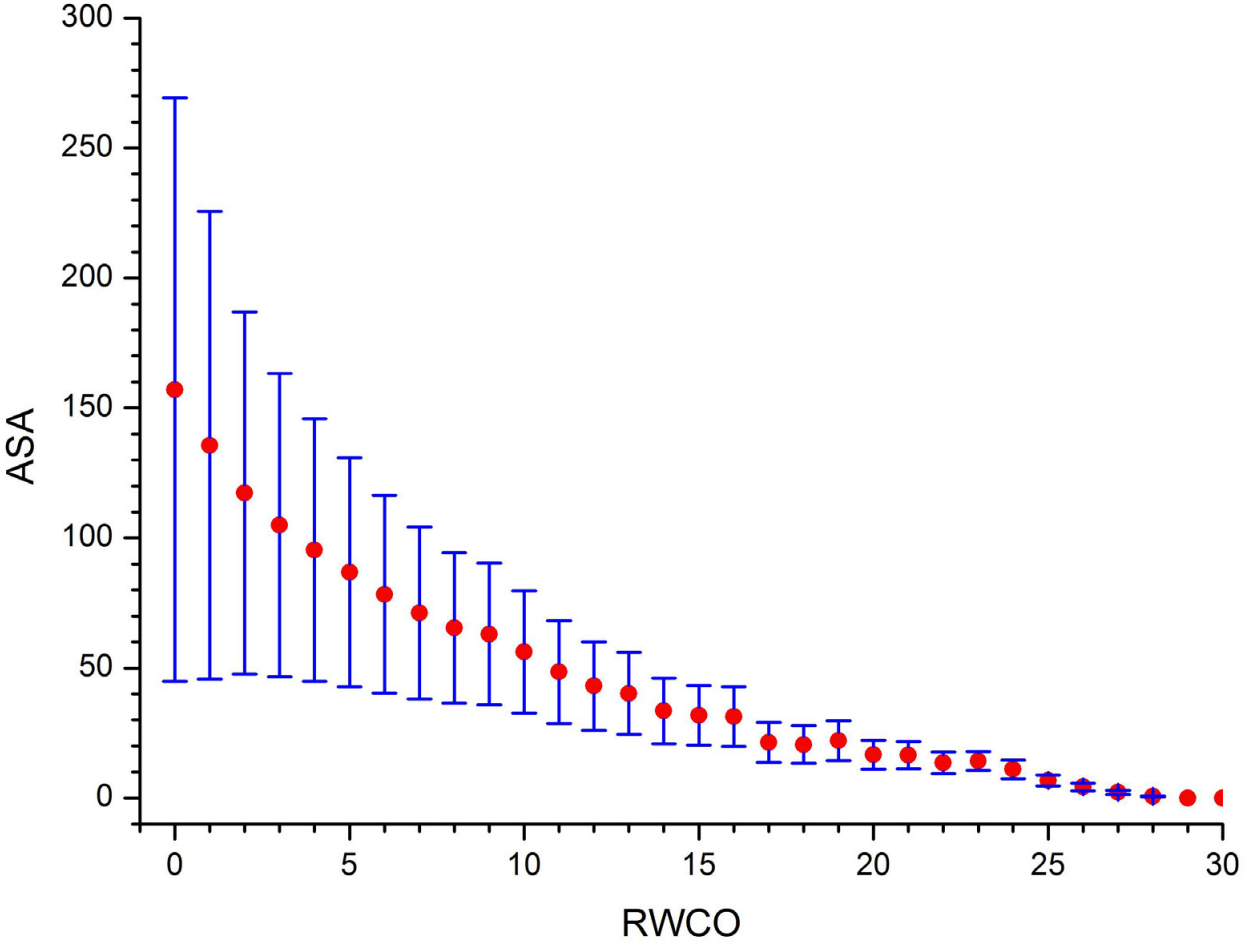
**The accessible surface area as a function of RWCO**. RWCO values are defined under the radius cutoff ***r***_***d ***_= 12 Å. Error bars represent the standard deviations (SD).

Since RWCO is a per-residue quantity of amino acid [11,12], it is natural to conjecture that there exists a relationship between RWCO and the solvent accessibility profile of amino acid residue. In order to investigate their connections, we extracted the accessible surface area (ASA) values of each residue in our dataset using the DSSP program [15]. The negative relationship between RWCO and ASA could be observed with a correlation coefficient of -0.463 (Figure 2). This means that the larger the ASA of a residue, the smaller the RWCO value of that residue, which is consistent with the expectation that the residue with small ASA has large numbers of contact residues in the structure space around itself.

### Predicting RWCO values using multiple sequence alignment profiles

As many studies have indicated, the evolutionary information implicitly contained in the multiple sequence alignments could provide better prediction performance compared with the single sequence alone. In this study, the position-specific scoring matrix (PSSM) generated by the PSI-BLAST program [31] served as the input to SVR. The important evolutionary information is stored in these multiple sequence alignment profiles. For an objective comparison with the results of Kinjo *et al*. [11,12], we also performed the same 15-fold cross-validation test in this study, i.e. 680 proteins were randomly divided into two parts: the training dataset with 630 proteins and the testing dataset with the remaining 50 proteins [11,12]. This procedure was repeated 15 times, generating the final 15 combinations of SVR training and testing datasets. At each cross-validation step, we built the SVR model using the normalized training set, predicted the normalized RWCO values using this model and then transformed to their absolute RWCO values. Four prediction performance measures the correlation coefficient (CC), root mean square error DevA_p_, RMSE_norm and RMSE_raw are given in Table 3 (column "LS").

**Table 3:** Correlation coefficients (CCs), Deviation and Root Mean Square Errors (RMSEs) for RWCO predictions using 15-fold cross-validations. The results are expressed as Mean ± Standard Deviation.

Performance	LS	LS+AA	LS+W	LS+SS	LS+W+AA	LS+W+SS	LS+W+AA+SS	LS+W+AA+SS_raw
CC	0.55 ± 0.02	0.56 ± 0.02	0.56 ± 0.02	0.58 ± 0.02	0.57 ± 0.02	0.59 ± 0.02	0.60 ± 0.02	0.58 ± 0.03
DevA_p_	0.94 ± 0.07	0.93 ± 0.06	0.92 ± 0.06	0.90 ± 0.07	0.91 ± 0.06	0.89 ± 0.06	0.87 ± 0.05	0.90 ± 0.07
RMSE_norm	0.82 ± 0.02	0.81 ± 0.02	0.80 ± 0.02	0.79 ± 0.02	0.79 ± 0.02	0.79 ± 0.02	0.78 ± 0.01	-
RMSE_raw	3.21 ± 0.07	3.18 ± 0.06	3.15 ± 0.06	3.10 ± 0.06	3.12 ± 0.06	3.07 ± 0.06	3.05 ± 0.06	3.09 ± 0.07

In the current work, RWCO is normalized using the entire benchmark dataset. More specifically, RWCO is normalized according to the formula (3) in the Methods Section using the Standard Deviation and mean raw RWCO values that are computed based on the whole dataset. We first computed the normalized RWCO values before SVR training and testing, then replaced the raw RWCO values by using these normalized values (both for the training and testing datasets). After predicting the normalized RWCO values for the test datasets, we restored the raw RWCO values by transforming the predicted normalized RWCO values to raw ones by using equation (3). The reason for using normalized RWCO values instead of the raw values here is that this strategy can improve the prediction performance and is more robust than if raw values are used. As suggested by the reviewer, we tested the predictive performance of the same sequence encoding scheme "LS+W+AA+SS" based on both the normalized values and raw ones, whose result comparison is shown in Table 3. It is clear that the predictor using normalized RWCO values is superior to that of using raw values- the CC improves from 0.58 to 0.60, whereas the values of DevAp and RMSE_raw drop from 0.90 to 0.87 and from 3.09 to 3.05, respectively. This normalization step is important for achieving better prediction performance in the training and testing SVR process.

It can be seen that the use of multiple sequence alignments for SVR training and testing yields CC = 0.55, DevA_p _= 0.94 and RMSE_raw = 3.21 (Table 3), which is already a statistically significant result. Although lower than other sequence encoding schemes with a CC less by about 0.05, using multiple sequence alignment in the form of PSI-BLAST profiles as input to SVR can still achieve a comparable prediction performance compared with other more complicated schemes. Thus implying that multiple sequence profiles contain essential information for accurately predicting RWCO values. This finding is also consistent with other studies such as predicting solvent accessibility [18,25], B factor profiles [19], contact numbers [14,20] and disulfide connectivity [27].

**Figure 3:**
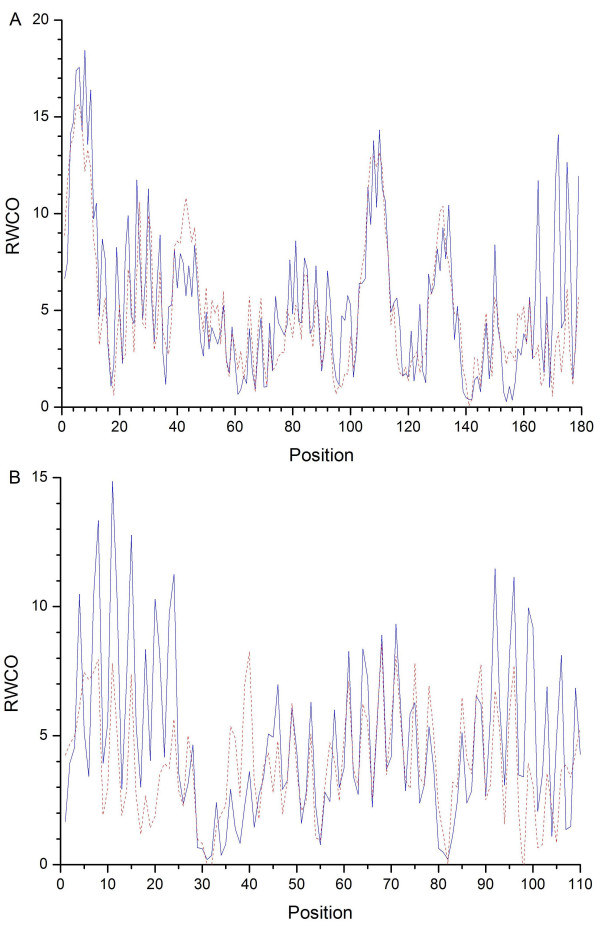
**The predicted and observed RWCO profiles for proteins integrin alpha-L (PDB code: 1mjn, chain A) and transcriptional activator GCN5 (PDB code: 1e6i, chain A)**. Predicted and observed RWCO values are represented by dashed red and solid blue lines, respectively. RWCO values are computed and predicted with a radius cutoff of 12 Å. (A) integrin alpha-L is predicted with a correlation coefficient of 0.81 and a root mean square error of 2.35; (B) Transcriptional activator GCN5 is predicted with a correlation coefficient of 0.61 and a root mean square error of 2.69.

For a better understanding of the CC and RMSE measures used here, we provided two prediction examples of well-predicted and badly-predicted proteins in Figure 3. This figure shows to what extent the predicted and observed RWCO values match each other. Protein integrin alpha-L (PDB: 1mjna) is well predicted with a CC of 0.81 and a RMSE of 2.35. The majority of the regions of this protein are in good agreement with its corresponding observed values, except several separate positions at the tail end. In contrast, the transcriptional activator GCN5 (PDB: 1e6ia) is poorly predicted with a CC of only 0.61 and an RMSE of 2.69.

**Figure 4:**
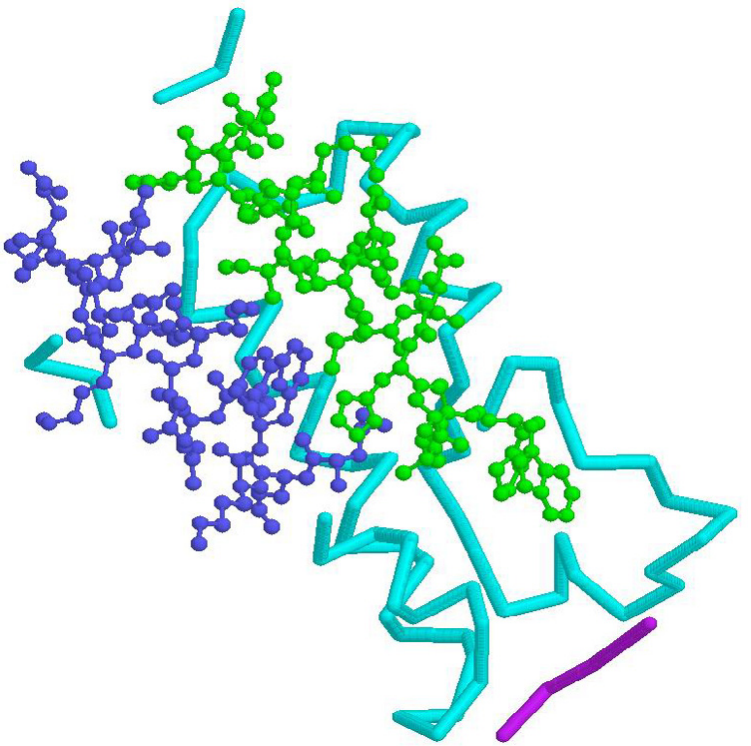
**The 3D structure of the predicted transcriptional activator GCN5 (PDB code: 1e6i, chain A), whose two badly predicted regions (from residue position 4 to 23 and from position 92 to 106) are highlighted using the green and blue ball-and-stick models, respectively**. The cyan backbone depicts other regions of the protein chain. This 3D molecular image was generated by the Protein Explorer program [36].

Figure 4 shows the 3D structure depiction of the badly-predicted transcriptional activator GCN5. It has two regions that are badly predicted: from residue position 4 to 23 (represented using the green ball-and-stick model) and from position 92 to 106 (represented using the blue ball-and-stick model). Possible reasons for explaining why some regions of a protein are not well predicted may be due to the fact that there are some residues with relatively small numbers in the dataset and therefore they are less adequately represented after input into SVR models. This would also account for the lower prediction accuracy in some regions of the protein. SVM usually achieves better prediction accuracy when using well-represented datasets than the inadequately-represented ones as the training and testing datasets.

### Improving the prediction performance by incorporating global information such as protein molecular weight and amino acid composition, as well as the predicted secondary structure by PSIPRED

The multiple sequence alignment profile used here is a kind of local sequence feature. However, we still need to take into account additional global features to further improve the prediction performance. Kinjo *et al*. also pointed out that the global context has an effect on the prediction accuracy and it might be useful to include more global features of amino acid sequences [12]. On the other hand, protein molecular weight, as another global sequence feature, could considerably improve the prediction accuracy [20]. We thus divided the protein sequences into four subgroups with equal protein numbers according to their molecular weights. We also incorporated the amino acid composition as the input vector to SVR.

In this work, we employed seven different encoding schemes, i.e. local sequence ("LS"), local sequence plus molecular weight ("LS+W"), local sequence plus amino acid composition ("LS+AA"), local sequence plus predicted secondary structure information by PSIPRED ("LS+SS"), local sequence together with molecular weight and amino acid composition ("LS+W+AA"), local sequence together with molecular weight and predicted secondary structure ("LS+W+SS"), local sequence, molecular weight, amino acid composition and predicted secondary structure information ("LS+W+AA+SS"). PSIPRED is a program to generate the probability profiles for three secondary structure state (helix, strand and coli) assignments for each residue of the predicted protein [31]. We extracted the 15 × 3 = 45 matrix from the output file of PSIPRED by selecting the sliding window size 15, and incorporated this matrix into the SVR model. For the last sequence encoding scheme "LS+W+AA+SS", a residue was encoded as a 15 × 20+1+20+15 × 3 = 366 dimensional vector. The prediction results for each subgroup are shown in Table 4.

**Table 4:** Correlation coefficients (CCs), Deviation and Root Mean Square Errors (RMSEs) for individual proteins in different protein weight groups.

Weight	Performance	LS	LS+AA	LS+W	LS+SS	LS+W+AA	LS+W+SS	LS+W+AA+SS
W≤ 12320	CC	0.52	0.53	0.54	0.58	0.55	0.59	0.60
	DevA_p_	0.92	0.91	0.89	0.87	0.87	0.84	0.84
	RMSE_norm	0.77	0.76	0.74	0.73	0.73	0.70	0.69
	RMSE_raw	3.02	2.97	2.89	2.85	2.86	2.75	2.74
12320<W≤ 17440	CC	0.57	0.58	0.59	0.60	0.60	0.61	0.62
	DevA_p_	0.91	0.90	0.88	0.88	0.87	0.86	0.85
	RMSE_norm	0.82	0.81	0.79	0.79	0.78	0.78	0.77
	RMSE_raw	3.21	3.18	3.12	3.10	3.09	3.01	2.99
17440<W≤ 26460	CC	0.57	0.58	0.58	0.59	0.58	0.60	0.60
	DevA_p_	0.92	0.92	0.92	0.89	0.92	0.89	0.89
	RMSE_norm	0.81	0.81	0.80	0.79	0.80	0.78	0.78
	RMSE_raw	3.17	3.16	3.15	3.08	3.14	3.06	3.05
W>26460	CC	0.52	0.52	0.53	0.53	0.53	0.53	0.55
	DevA_p_	1.00	0.99	0.98	0.99	0.96	0.98	0.94
	RMSE_norm	0.88	0.87	0.88	0.87	0.87	0.87	0.86
	RMSE_raw	3.44	3.42	3.45	3.40	3.42	3.42	3.39
All	CC	0.55	0.56	0.56	0.58	0.57	0.59	0.60
	DevA_p_	0.94	0.93	0.92	0.90	0.91	0.89	0.87
	RMSE_norm	0.82	0.81	0.80	0.79	0.79	0.79	0.78
	RMSE_raw	3.21	3.18	3.15	3.10	3.12	3.07	3.05

As a kind of global feature using either the amino acid composition ("LS+AA") or protein molecular weight ("LS+W") yields the better prediction performance compared with local sequence alone. However, in contrast to amino acid composition, it is worth noting that protein molecular weight here can give a more significant improvement. The significance of molecular weight on the prediction performance has been previously observed in the prediction study of protein contact numbers [20]. This effect is even remarkable when predicting proteins with relatively small molecular weights. For instance, for proteins with weights less than 12320 Daltons, "LS+AA" schemes can give prediction accuracy with a CC of 0.53, DevA_p _of 0.91 and RMSE_raw of 2.97, while "LS+W" can increase the CC to 0.54 and decrease DevA_p _and RMSE_raw to 0.89 and 2.89, respectively. Furthermore, when combining the amino acid and molecular weight information, there is still a significant improvement in the final prediction performance. The encoding scheme "LS+W+AA" could predict RWCO values with an overall CC of 0.57, DevA_p _of 0.91 and RMSE_raw of 3.12.

Proteins with relatively large molecular weights are less well predicted than proteins with smaller molecular weights. For example, for proteins with molecular weights larger than 26460 Daltons, the "LS" encoding scheme could only predict their RWCO values with a CC of 0.52, DevA_p _of 1.00 and RMSE_raw of 3.44, which is rather lower than for the other protein groups. Even after adopting the "LS+W+AA+SS" encoding scheme, the resulting improvement is still not as significant as other protein groups, i.e. with a CC of 0.55, DevA_p _of 0.94 and RMSE_raw of 3.39. This might be attributable to the small numbers of large proteins in the current datasets which are under-represented when building SVR models, while the availability of the training samples could in turn affect the predictive ability of built SVM models to a large extent.

When compared with the global features such as amino acid composition ("AA") and protein molecular weight ("W"), however, the predicted secondary structure by PSIPRED seems to be the most important determinant of our predictors. This is apparent by observing the significant performance improvement that the CC increases from 0.55 using the "LS" encoding scheme to 0.58 using the "LS+SS" scheme, whereas the values of DevAp, RMSE_norm and RMSE_raw decreases from 0.94, 0.82, and 3.21 to 0.90, 0.79 and 3.10, respectively. We can also draw the same conclusion by comparing the performance improvement of the "LS+W" encoding scheme with that of the "LS+W+SS" scheme. The CC improves from 0.56 to 0.59, while the DevAp and RMSE_raw values decrease from 0.92 to 0.89 and 3.15 to 3.07, respectively, after incorporating "SS" information in the encoding scheme "LS+W". As a result, our method achieved the overall best prediction accuracy after adopting the encoding scheme "LS+W+AA+SS", i.e. combing all the four kinds of information. The average CC, DevA_p _and RMSE_raw scores are 0.60, 0.87 and 3.05, respectively.

**Figure 5:**
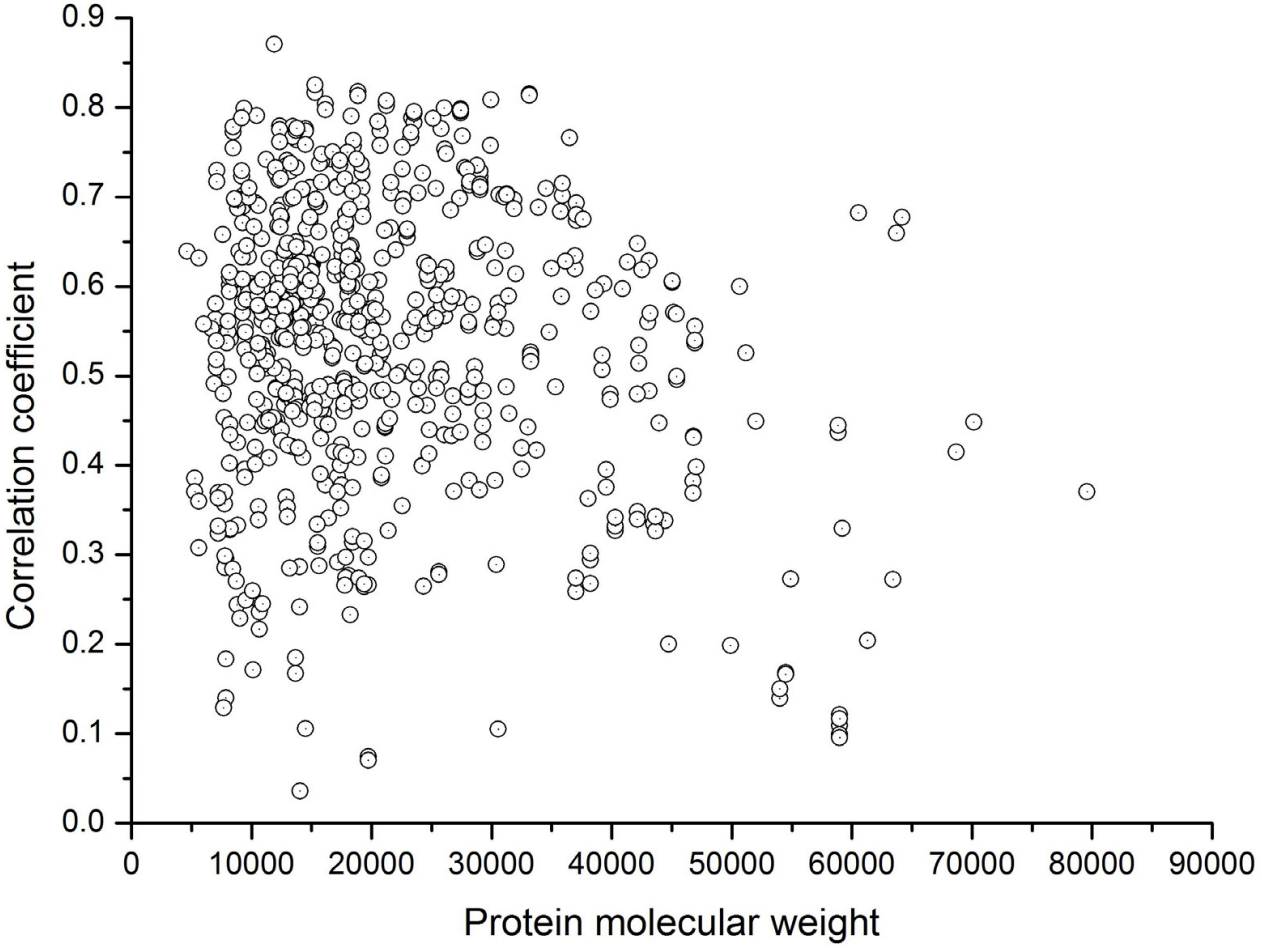
**The CC distribution versus protein molecular weight**. Each circle in the plot represents a protein sequence in the testing datasets.

To visualize the prediction accuracy of individual protein, we plotted the CC against the corresponding protein molecular weight in Figure 5. It can be shown that most proteins tested are situated in the region with CCs larger than 0.4 or more, while there also exist some separate proteins which are poorly predicted with CCs lower than 0.3. These proteins that are poorly predicted are found to be mainly distributed on both sides of protein weights, suggesting that both some small and large proteins are less accurately predicted.

**Figure 6:**
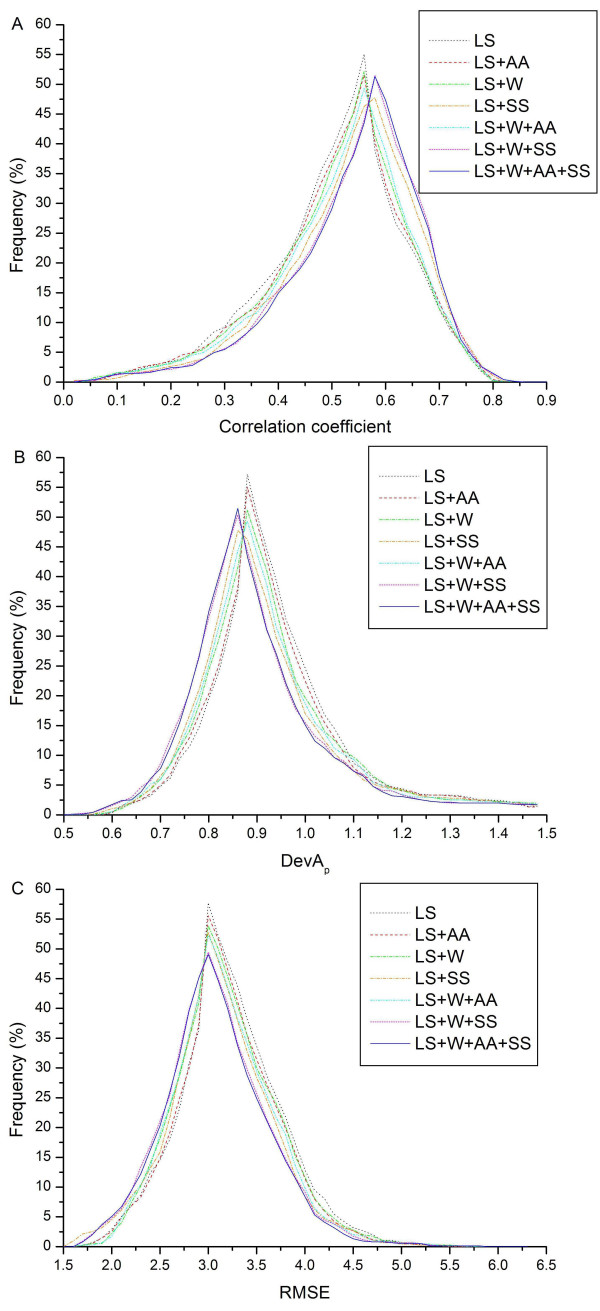
**The distributions of CC, DevA_p _and RMSE for the seven different encoding schemes**. "LS", "LS+AA", "LS+W", "LS+SS", "LS+W+AA", "LS+W+SS", and "LS+W+AA+SS" are represented by dotted black, dashed red, dot-and-dashed green, dot-and-dashed orange, dash-dot-and-dotted cyan, short-dashed magenta and solid blue lines, respectively.

We also calculated the overall distributions of CC, DevA_p _and RMSE of the testing proteins sequences for the seven different encoding schemes, which are depicted in Figure 6. The peak values of CC, DevA_p _and RMSE are close to 0.60, 0.86 and 3.0, respectively. For the CC distribution, the rightmost curve in the plot represents the best prediction method, while for DevA_p _and RMSE distributions, the leftmost curves denote the best method. All the three distributions of CC, DevA_p _and RMSE indicated that the "LS+W+AA+SS" encoding scheme leads to the best performance.

**Figure 7:**
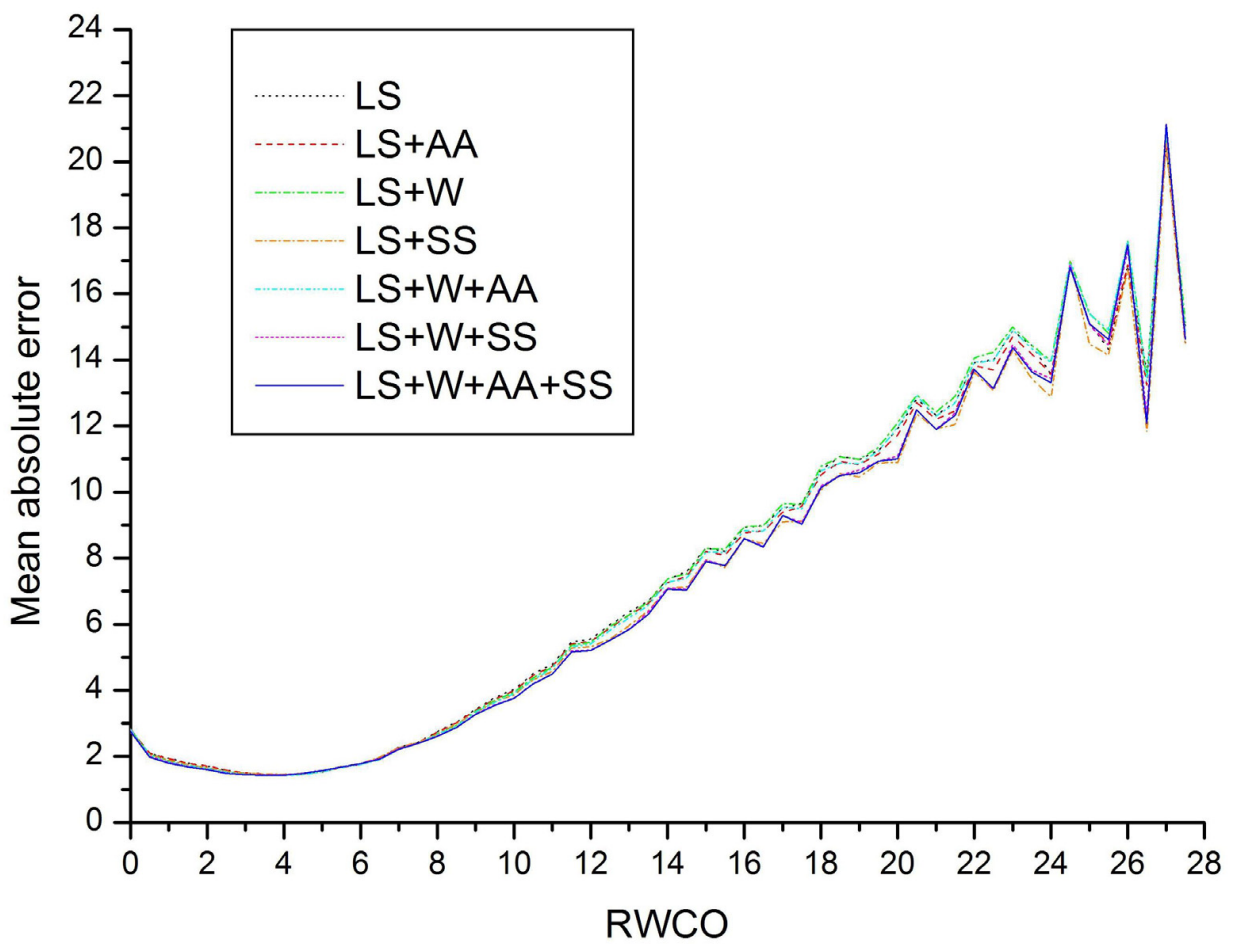
**The mean absolute errors for different RWCO values of residues**. The seven different encoding schemes "LS", "LS+AA", "LS+W", "LS+SS", "LS+W+AA", "LS+W+SS", and "LS+W+AA+SS" are represented by dotted black, dashed red, dot-and-dashed green, dot-and-dashed orange, dash-dot-and-dotted cyan, short-dashed magenta and solid blue lines, respectively.

The mean absolute errors (MAEs) of residues with different RWCO values are plotted in Figure 7. It can be seen that the "LS+W+AA+SS" encoding scheme leads to the least mean absolute error for the majority of the regions and thus could give the best prediction performance. Residues with RWCO value 4 are predicted with the least mean absolute errors, due to the fact that these proteins have the largest numbers in the current dataset and thus are adequately represented. On the other hand, residues with larger RWCO values (RWCO>20) have larger MAEs and are thus worst predicted.

### Comparison with other methods

We also compared our SVR based method with other prediction methods, such as the linear regression method [11] and critical random networks (CRNs) [12]. For an objective comparison, these methods are all measured on the same training and testing datasets using 15-fold cross-validation. The results are summarized in Table 5.

**Table 5:** Comparison of predictive performance with different approaches. The results were obtained by 15-fold cross-validation.

Methods	Prediction accuracy (%)			
	
	CC	DevA_p_	RMSE_norm	RMSE_raw
Support vector regression	0.60	0.87	0.78	3.05
Linear regression	0.59	1.03	-	-
Critical random network	0.60	0.88	-	-

-The result can not be obtained from the relevant papers.

When selecting the sequence encoding scheme "LS+W+AA+SS", the SVR method could achieve the best prediction accuracy with a CC of 0.60, DevA_p _of 0.87, and RMSE_raw of 3.05. The linear regression method is based on the simple linear regression scheme and achieved prediction accuracy with a CC of 0.59 and DevA_p _of 1.03 [11]. CRN predicted RWCO values by defining a linear function of a state vector associated with a target sequence, namely, the position-specific scoring matrices (PSSMs) generated from PSI-BLAST and achieved a best prediction performance with CC of 0.60 and DevA_p _of 0.88 [12]. Both linear regression and CRN methods employed the same local window size of 52 residues to achieve their respective best performance. As can be seen, the SVR method performed much better than the simple linear regression method and slightly better than that of the CRN method with the same accuracy of CC and smaller DevA_p _values. These results suggest that the SVR method is at least competitive with, if not better than, the previously developed methods.

## Discussion

Residue-wise contact order, in conjunction with secondary structure, solvent accessibility, B factors and contact number, can provide complementary and indispensable information for the ultimate prediction of protein three-dimensional structures. Due to the importance of residue contact orders on the protein folding and protein structure prediction, studies in this direction are receiving more and more attention recently [8-13].

Several ways may help to further improve the prediction performance in the future. The first approach is to use more accurately determined PDB structures with better resolutions. The second is to incorporate other informative and complementary features, such as protein solvent accessibility and contact numbers, which have been proved to have high correlations with RWCO values in proteins [11,12]. The third strategy can focus on how to effectively represent those under-represented proteins with lower or higher molecular weights. Increasing the ratio of these proteins in the whole dataset could also contribute to enhancing the prediction accuracy. Further improvement is possibly achieved by using refined datasets and combining more informative multiple feature descriptors together.

As a new machine learning method, support vector regression has many attractive features and our study presented here has further enhanced its useful application in reliably predicting residue-wise contact orders in proteins. The present method may also be useful in protein structure prediction, protein folding rate prediction and protein engineering applications.

## Conclusion

In this paper, we have developed a novel approach to predict the residue-wise contact order in proteins using support vector regression based on the local protein sequence descriptor (multiple sequence alignments in the form of PSI-BLAST profiles) and two global descriptors (protein molecular weight and amino acid composition). The predicted secondary structure by PSIPRED also served as input to the SVR. For completeness, we introduced seven different sequence encoding combinations and investigated their effects on the prediction performance. We found that using the local sequence descriptor could provide benchmark prediction accuracy with a CC of 0.55, DevA_p _of 0.94 and RMSE of 3.21. Furthermore, after adopting the sequence encoding scheme "LS+W+AA+SS" that combined the local sequence descriptor, global descriptors and the predicted secondary structure together, our method could yield the best prediction performance with a CC of 0.60, DevA_p _of 0.87 and RMSE of 3.05, a significant improvement over the accuracy based on local sequence information alone. Our results indicated that both the local sequence context and the predicted secondary structure are important determinants in predicting residue-wise contact orders in proteins. We have demonstrated that the SVR approach is competitive with other existing algorithms based on linear regression models. Due to its attractive potential in condensing information and regressing value profiles, it is anticipated that the SVR method will play a more important role in analyzing large-scale genome and proteome data as more biological data becomes available through genome sequencing projects.

## Methods

### Dataset

We used the same dataset previously prepared by Kinjo and Nishikawa [11,12], which included 680 protein sequences and was originally extracted from ASTRAL database version 1.65 [16]. This is a well-defined dataset and each of the protein chains represents a superfamily from all-α, all-β, α/β, α+β and Multi-domain proteins in SCOP database [17]. The sequence identity between each pair of chains was less than 40%.

However, in the current ASTRAL SCOP version 1.69 (generated on August 1, 2005), some original protein chains included in version 1.65 are replaced or discarded. They are d1dj0a1 (replaced by d1dj0a_), d1dkza_ (replaced by d1dkza1 and d1dkza2), d1fvka1 (replaced by d1fvka_), d1gdoa (replaced by d1xffa_), d1hf8a_ (replaced by d1hf8a1 and d1hf8a2), d1jx4a_ (replaced by d1jx4a1 and d1jx4a2), and d1oi2a1 (replaced by d1oi2a_). In order to compare our results with those of Kinjo *et al*., we simply restored these seven original entries in version 1.65 instead of the updated ones.

There are a total of 120421 residues in this dataset. The protein chain names and their corresponding amino acid sequences can be found in the Additional File 1 (supplementary material). The detailed RWCO information with a radius cutoff of 12Å can be found in the Additional File 2 (supplementary material).

### Residue-wise contact order

The concept of residue-wise contact order (RWCO) was first introduced by Kinjo and Nishikawa [11,12]. The discrete RWCO values of the *i*-th residue in a protein sequence with *M *residues is defined by

RWCOi=1M∑j:|j−i|>2M|i−j|σ(ri,j){σ(ri,j)=1,if ri,j<rdσ(ri,j)=0,if ri,j≥rd,     (1)
 MathType@MTEF@5@5@+=feaafiart1ev1aaatCvAUfKttLearuWrP9MDH5MBPbIqV92AaeXatLxBI9gBaebbnrfifHhDYfgasaacH8akY=wiFfYdH8Gipec8Eeeu0xXdbba9frFj0=OqFfea0dXdd9vqai=hGuQ8kuc9pgc9s8qqaq=dirpe0xb9q8qiLsFr0=vr0=vr0dc8meaabaqaciaacaGaaeqabaqabeGadaaakeaafaqabeqacaaabaGaemOuaiLaem4vaCLaem4qamKaem4ta80aaSbaaSqaaiabdMgaPbqabaGccqGH9aqpdaWcaaqaaiabigdaXaqaaiabd2eanbaadaaeWbqaaiabcYha8jabdMgaPjabgkHiTiabdQgaQjabcYha8HGaciab=n8aZnaabmGabaGaemOCai3aaSbaaSqaaiabdMgaPjabcYcaSiabdQgaQbqabaaakiaawIcacaGLPaaaaSqaaiabdQgaQjabcQda6iabcYha8jabdQgaQjabgkHiTiabdMgaPjabcYha8jabg6da+iabikdaYaqaaiabd2eanbqdcqGHris5aaGcbaWaaiqabeaafaqabeGabaaabaGae83Wdm3aaeWaceaacqWGYbGCdaWgaaWcbaGaemyAaKMaeiilaWIaemOAaOgabeaaaOGaayjkaiaawMcaaiabg2da9iabigdaXiabcYcaSiabdMgaPjabdAgaMjabbccaGiabdkhaYnaaBaaaleaacqWGPbqAcqGGSaalcqWGQbGAaeqaaOGaeyipaWJaemOCai3aaSbaaSqaaiabdsgaKbqabaaakeaacqWFdpWCdaqadiqaaiabdkhaYnaaBaaaleaacqWGPbqAcqGGSaalcqWGQbGAaeqaaaGccaGLOaGaayzkaaGaeyypa0JaeGimaaJaeiilaWIaemyAaKMaemOzayMaeeiiaaIaemOCai3aaSbaaSqaaiabdMgaPjabcYcaSiabdQgaQbqabaGccqGHLjYScqWGYbGCdaWgaaWcbaGaemizaqgabeaakiabcYcaSaaaaiaawUhaaaaacaWLjaGaaCzcamaabmGabaGaeGymaedacaGLOaGaayzkaaaaaa@8A38@

where *r*_*i*,*j *_is the distance between the *C_β _*atoms of the *i*-th and *j*-th residues (*C_α _*atoms for glycine) in the protein sequence. Two residues are considered to be in contact if their *C_β _*atoms locate within a sphere of the threshold radius *r*_*d*_. Note that the trivial contacts between the nearest and second-nearest residues are excluded. In order to smooth the discrete RWCO values, Kinjo *et al*. proposed a particular sigmoid function [11,12,14], which is given by

σ(*r_i, j_*) = 1/{1+exp[*w*(*r_i, j _*- *r_d_*)]},     (2)

where *w *is a parameter that determines the sharpness of the sigmoid function. In the present study, for the sake of comparison, we set ***r***_***d ***_= 12 Å and *w *= 3, which was adopted by Kinjo *et al*. [11,12].

### Normalization of RWCO

Previous studies have indicated that using normalized values can lead to better and more stable prediction performance compared with the raw values [18-20]. The normalized RWCO value is given by

yi=y′i−y¯SD,     (3)
 MathType@MTEF@5@5@+=feaafiart1ev1aaatCvAUfKttLearuWrP9MDH5MBPbIqV92AaeXatLxBI9gBaebbnrfifHhDYfgasaacH8akY=wiFfYdH8Gipec8Eeeu0xXdbba9frFj0=OqFfea0dXdd9vqai=hGuQ8kuc9pgc9s8qqaq=dirpe0xb9q8qiLsFr0=vr0=vr0dc8meaabaqaciaacaGaaeqabaqabeGadaaakeaacqWG5bqEdaWgaaWcbaGaemyAaKgabeaakiabg2da9maalaaabaGafmyEaKNbauaadaWgaaWcbaGaemyAaKgabeaakiabgkHiTiqbdMha5zaaraaabaGaem4uamLaemiraqeaaiabcYcaSiaaxMaacaWLjaWaaeWaceaacqaIZaWmaiaawIcacaGLPaaaaaa@3D49@

where *y*_*i *_is the normalized RWCO value of *i *residue, y′i
 MathType@MTEF@5@5@+=feaafiart1ev1aaatCvAUfKttLearuWrP9MDH5MBPbIqV92AaeXatLxBI9gBaebbnrfifHhDYfgasaacH8akY=wiFfYdH8Gipec8Eeeu0xXdbba9frFj0=OqFfea0dXdd9vqai=hGuQ8kuc9pgc9s8qqaq=dirpe0xb9q8qiLsFr0=vr0=vr0dc8meaabaqaciaacaGaaeqabaqabeGadaaakeaacuWG5bqEgaqbamaaBaaaleaacqWGPbqAaeqaaaaa@2FBA@ is the raw RWCO value, y¯
 MathType@MTEF@5@5@+=feaafiart1ev1aaatCvAUfKttLearuWrP9MDH5MBPbIqV92AaeXatLxBI9gBaebbnrfifHhDYfgasaacH8akY=wiFfYdH8Gipec8Eeeu0xXdbba9frFj0=OqFfea0dXdd9vqai=hGuQ8kuc9pgc9s8qqaq=dirpe0xb9q8qiLsFr0=vr0=vr0dc8meaabaqaciaacaGaaeqabaqabeGadaaakeaacuWG5bqEgaqeaaaa@2E3F@ is the mean raw RWCO value, and SD is the standard deviation.

Following the same strategy in predicting the B-factor and contact number [19,20], we first predicted the normalized RWCO values from amino acid sequences, and then recovered the absolute RWCO values from the predicted normalized values using the above equation (3).

### Support vector regression

Support vector machine (SVM) is a new machine learning method based on the structural risk minimization in Statistical Learning Theory (SLT) and has been successfully applied to a wide range of pattern recognition problems, including microarray data analysis [21], protein secondary structure prediction [5], protein subcellular localization prediction [22-24], protein solvent accessibility prediction [25], proline *cis*/*trans *isomerization prediction [26], disulfide connectivity prediction [27] and DNA-binding site prediction [28]. More detailed description of SVM can be found in Vapnik's publications [29,30]. SVM has two practical modes: the classification mode (support vector classification, SVC) and regression mode (support vector regression, SVR). In contrast to SVC, SVR has an outstanding ability in predicting the raw values of the tested samples. It is especially effective when the input data is characterized by high dimension and non-linear function. As a novel machine learning method, SVR has been successfully applied in computational biology to predicting accessible surface areas [18], protein B factors [19], contact numbers [20], estimating missing value in microarray data [32], predicting gene expression level [33], and peptide-MHC binding affinities [34]. In this study, we describe the first use of an SVR approach to predict RWCO values from the primary amino acid sequences.

To find the function between the protein sequence and the normalized RWCO values, we use ε-insensitive support vector regression (ε-SVR) [29,30]. The objective of the regression problem is to estimate an unknown continuous-valued function *y *= *f *(*x*), which is based on a finite number of samples [18,19]. Let {(*x*_*i*_, *y*_*i*_)} (*i *= 1, ..., *M*) denote a set of training data, where feature vector *x*_*i *_denotes residue *i *in a protein sequence with *M *residues, and *y*_*i *_represents its corresponding normalized RWCO value.

The expected function of SVR is

*f*(*x_i_*) = ⟨*W*, (Φ*x_i_*)⟩ + *b*.     (4)

Here, *W *is the weight, *b *is the bias, and ⟨*W*, (Φ*x_i_*)⟩ is the inner product of *W *and Φ(*x*_*i*_). To estimate the function f(x), two slack variables *ζ_i _*and ξi∗
 MathType@MTEF@5@5@+=feaafiart1ev1aaatCvAUfKttLearuWrP9MDH5MBPbIqV92AaeXatLxBI9gBaebbnrfifHhDYfgasaacH8akY=wiFfYdH8Gipec8Eeeu0xXdbba9frFj0=OqFfea0dXdd9vqai=hGuQ8kuc9pgc9s8qqaq=dirpe0xb9q8qiLsFr0=vr0=vr0dc8meaabaqaciaacaGaaeqabaqabeGadaaakeaaiiGacqWF+oaEdaqhaaWcbaGaemyAaKgabaGaey4fIOcaaaaa@30ED@ are introduced to measure the deviation of samples outside the ε-insensitive tube. Thus the optimization problem of SVR can be expressed as

minimize12‖W‖2+C∑i=1M(ξi+ξi∗),     (5)
 MathType@MTEF@5@5@+=feaafiart1ev1aaatCvAUfKttLearuWrP9MDH5MBPbIqV92AaeXatLxBI9gBaebbnrfifHhDYfgasaacH8akY=wiFfYdH8Gipec8Eeeu0xXdbba9frFj0=OqFfea0dXdd9vqai=hGuQ8kuc9pgc9s8qqaq=dirpe0xb9q8qiLsFr0=vr0=vr0dc8meaabaqaciaacaGaaeqabaqabeGadaaakeaafaqabeqacaaabaGaeeyBa0MaeeyAaKMaeeOBa4MaeeyAaKMaeeyBa0MaeeyAaKMaeeOEaONaeeyzaugabaWaaSaaaeaacqaIXaqmaeaacqaIYaGmaaWaauWaceaacqWGxbWvaiaawMa7caGLkWoadaahaaWcbeqaaiabikdaYaaakiabgUcaRiabdoeadnaaqahabaWaaeWaceaaiiGacqWF+oaEdaWgaaWcbaGaemyAaKgabeaakiabgUcaRiab=57a4naaDaaaleaacqWGPbqAaeaacqGHxiIkaaaakiaawIcacaGLPaaaaSqaaiabdMgaPjabg2da9iabigdaXaqaaiabd2eanbqdcqGHris5aOGaeiilaWcaaiaaxMaacaWLjaWaaeWaceaacqaI1aqnaiaawIcacaGLPaaaaaa@568F@

subject to{f(xi)−yi≤ε+ξiyi−f(xi)≤ε+ξi∗ξi,ξi∗≥0,i=1,...,M,     (6)
 MathType@MTEF@5@5@+=feaafiart1ev1aaatCvAUfKttLearuWrP9MDH5MBPbIqV92AaeXatLxBI9gBaebbnrfifHhDYfgasaacH8akY=wiFfYdH8Gipec8Eeeu0xXdbba9frFj0=OqFfea0dXdd9vqai=hGuQ8kuc9pgc9s8qqaq=dirpe0xb9q8qiLsFr0=vr0=vr0dc8meaabaqaciaacaGaaeqabaqabeGadaaakeaafaqabeqacaaabaGaee4CamNaeeyDauNaeeOyaiMaeeOAaOMaeeyzauMaee4yamMaeeiDaqNaeeiiaaIaeeiDaqNaee4Ba8gabaWaaiqabeaafaqabeWabaaabaGaemOzay2aaeWaceaacqWG4baEdaWgaaWcbaGaemyAaKgabeaaaOGaayjkaiaawMcaaiabgkHiTiabdMha5naaBaaaleaacqWGPbqAaeqaaOGaeyizImkcciGae8xTduMaey4kaSIae8NVdG3aaSbaaSqaaiabdMgaPbqabaaakeaacqWG5bqEdaWgaaWcbaGaemyAaKgabeaakiabgkHiTiabdAgaMnaabmGabaGaemiEaG3aaSbaaSqaaiabdMgaPbqabaaakiaawIcacaGLPaaacqGHKjYOcqWF1oqzcqGHRaWkcqWF+oaEdaqhaaWcbaGaemyAaKgabaGaey4fIOcaaaGcbaGae8NVdG3aaSbaaSqaaiabdMgaPbqabaGccqGGSaalcqWF+oaEdaqhaaWcbaGaemyAaKgabaGaey4fIOcaaOGaeyyzImRaeGimaaJaeiilaWIaemyAaKMaeyypa0JaeGymaeJaeiilaWIaeiOla4IaeiOla4IaeiOla4IaeiilaWIaemyta0KaeiilaWcaaaGaay5EaaaaaiaaxMaacaWLjaWaaeWaceaacqaI2aGnaiaawIcacaGLPaaaaaa@786C@

where *C *is the regulation parameter that controls the trade off between the margin and prediction error denoted by the slack variables *ζ_i _*and ξi∗
 MathType@MTEF@5@5@+=feaafiart1ev1aaatCvAUfKttLearuWrP9MDH5MBPbIqV92AaeXatLxBI9gBaebbnrfifHhDYfgasaacH8akY=wiFfYdH8Gipec8Eeeu0xXdbba9frFj0=OqFfea0dXdd9vqai=hGuQ8kuc9pgc9s8qqaq=dirpe0xb9q8qiLsFr0=vr0=vr0dc8meaabaqaciaacaGaaeqabaqabeGadaaakeaaiiGacqWF+oaEdaqhaaWcbaGaemyAaKgabaGaey4fIOcaaaaa@30ED@.

The final regression function can be formulated as

f(x)=∑i=1M(αi−αi∗)K(xi,x)+b,     (7)
 MathType@MTEF@5@5@+=feaafiart1ev1aaatCvAUfKttLearuWrP9MDH5MBPbIqV92AaeXatLxBI9gBaebbnrfifHhDYfgasaacH8akY=wiFfYdH8Gipec8Eeeu0xXdbba9frFj0=OqFfea0dXdd9vqai=hGuQ8kuc9pgc9s8qqaq=dirpe0xb9q8qiLsFr0=vr0=vr0dc8meaabaqaciaacaGaaeqabaqabeGadaaakeaacqWGMbGzdaqadiqaaiabdIha4bGaayjkaiaawMcaaiabg2da9maaqahabaWaaeWaceaaiiGacqWFXoqydaWgaaWcbaGaemyAaKgabeaakiabgkHiTiab=f7aHnaaDaaaleaacqWGPbqAaeaacqGHxiIkaaaakiaawIcacaGLPaaacqWGlbWsdaqadiqaaiabdIha4naaBaaaleaacqWGPbqAaeqaaOGaeiilaWIaemiEaGhacaGLOaGaayzkaaGaey4kaSIaemOyaigaleaacqWGPbqAcqGH9aqpcqaIXaqmaeaacqWGnbqta0GaeyyeIuoakiabcYcaSiaaxMaacaWLjaWaaeWaceaacqaI3aWnaiaawIcacaGLPaaaaaa@517C@

where *a*_*i *_and αi∗
 MathType@MTEF@5@5@+=feaafiart1ev1aaatCvAUfKttLearuWrP9MDH5MBPbIqV92AaeXatLxBI9gBaebbnrfifHhDYfgasaacH8akY=wiFfYdH8Gipec8Eeeu0xXdbba9frFj0=OqFfea0dXdd9vqai=hGuQ8kuc9pgc9s8qqaq=dirpe0xb9q8qiLsFr0=vr0=vr0dc8meaabaqaciaacaGaaeqabaqabeGadaaakeaaiiGacqWFXoqydaqhaaWcbaGaemyAaKgabaGaey4fIOcaaaaa@30C9@ are Lagrange multipliers, and the kernel function *K*(*x*_*i*_, *x*) = ⟨ Φ(*x_i_*), (*x*)⟩ Only the non-zero values of the Lagrange multipliers contribute to the ultimate SVR prediction, whose associated samples are known as support vectors. As a contrast, those zero-valued Lagrange multipliers falling inside the ε-insensitive tube make no contribution to the regression. Normally, the number of support vectors is much smaller than that of the samples, thus SVR has the attractive property of condensing information in the training samples which is represented by these useful support vectors with non-zero values.

The kernel function *K *(*x*_*i*_, *x*) has several different forms, such as polynomial kernel function, radial basis kernel function (RBF), sigmoid kernel function, etc. The radial basis kernel function is adopted in this study, which is given by

*K*(*x_i_*, *x*) = exp(-*γ *||*x_i _*- *x*||^2^),     (8)

where γ parameter needs to be regulated.

We used SVM_light, an implementation of Vapnik's SVM for support vector classification, regression and pattern recognition [35]. In the present study, we selected radial basis kernel function at ε = 0.01, γ = 0.01 and *C *= 5.0 to build the SVR models. This combination of parameters has been proven to yield the best performance in previous studies of predicting accessible surface area, B-factor and contact number [18-20].

### Sequence encoding scheme

Since numerous studies have well established that the prediction performance resulting from using multiple sequence alignments in the form of PSI-BLAST [31] profiles usually outperforms that of single sequence [11,12,18-20,25-28], we are more interested in utilizing multiple sequence encoding schemes here, in which the intermediate PSI-BLAST generated position-specific scoring matrix (PSSM) is used as the direct input to SVR.

We extracted the local sequence fragments of the centered residues of interest by a sliding window coding scheme, with window length 2*l*+1, where *l *is the half window size. We ran blastpgp program in the PSI-BLAST package to query each protein in the dataset against the NCBI nr database to generate the PSSM profiles, by three iterations of PSI-BLAST, with a cutoff *E*-value of 10^-7^. The PSSM is an *M *× 20 matrix, where *M *is the target sequence length and 20 is the number of amino acid types. Each element of the matrix represents the log-likelihood for each residue position in the multiple sequence alignment. Evolutionary information was included in this window as the input information coded by *M *× 20 dimensional vectors.

For the sake of SVR input and process, we simply divided all the elements in the PSSM profiles by 10 to normalize them, thus most values fell between -1.0 and 1.0. We selected a windows size of M = 15 to build the SVR predictors, which has been proven to yield the best performance in previous studies [18-20].

### Prediction performance evaluation

In order to objectively evaluate the prediction performance of our approach, we employed the 15-fold cross-validation methods. The 680 protein sequences used in this study were randomly divided into fifteen subsets with roughly equal numbers of protein sequences. In each validation step, one subset was singled out in turn as the testing dataset, while the rest were used as the training dataset.

To measure the performance of SVR methods in this application, we calculated the Pearson's correlation coefficients (CC) between the predicted and observed RWCO values in a protein sequence as given by

CC=∑i=1N(xi−x¯)(yi−y¯)[∑i=1N(xi−x¯)2][∑i=1N(yi−y¯)2],     (9)
 MathType@MTEF@5@5@+=feaafiart1ev1aaatCvAUfKttLearuWrP9MDH5MBPbIqV92AaeXatLxBI9gBaebbnrfifHhDYfgasaacH8akY=wiFfYdH8Gipec8Eeeu0xXdbba9frFj0=OqFfea0dXdd9vqai=hGuQ8kuc9pgc9s8qqaq=dirpe0xb9q8qiLsFr0=vr0=vr0dc8meaabaqaciaacaGaaeqabaqabeGadaaakeaacqWGdbWqcqWGdbWqcqGH9aqpdaWcaaqaamaaqahabaWaaeWaceaacqWG4baEdaWgaaWcbaGaemyAaKgabeaakiabgkHiTiqbdIha4zaaraaacaGLOaGaayzkaaWaaeWaceaacqWG5bqEdaWgaaWcbaGaemyAaKgabeaakiabgkHiTiqbdMha5zaaraaacaGLOaGaayzkaaaaleaacqWGPbqAcqGH9aqpcqaIXaqmaeaacqWGobGta0GaeyyeIuoaaOqaamaakaaabaWaamWaceaadaaeWbqaamaabmGabaGaemiEaG3aaSbaaSqaaiabdMgaPbqabaGccqGHsislcuWG4baEgaqeaaGaayjkaiaawMcaamaaCaaaleqabaGaeGOmaidaaaqaaiabdMgaPjabg2da9iabigdaXaqaaiabd6eaobqdcqGHris5aaGccaGLBbGaayzxaaWaamWaceaadaaeWbqaamaabmGabaGaemyEaK3aaSbaaSqaaiabdMgaPbqabaGccqGHsislcuWG5bqEgaqeaaGaayjkaiaawMcaamaaCaaaleqabaGaeGOmaidaaaqaaiabdMgaPjabg2da9iabigdaXaqaaiabd6eaobqdcqGHris5aaGccaGLBbGaayzxaaaaleqaaaaakiabcYcaSiaaxMaacaWLjaWaaeWaceaacqaI5aqoaiaawIcacaGLPaaaaaa@6B5E@

where *x*_*i *_and *y*_*i *_are the observed and predicted normalized RWCO values of the *i*-th residue, and x¯
 MathType@MTEF@5@5@+=feaafiart1ev1aaatCvAUfKttLearuWrP9MDH5MBPbIqV92AaeXatLxBI9gBaebbnrfifHhDYfgasaacH8akY=wiFfYdH8Gipec8Eeeu0xXdbba9frFj0=OqFfea0dXdd9vqai=hGuQ8kuc9pgc9s8qqaq=dirpe0xb9q8qiLsFr0=vr0=vr0dc8meaabaqaciaacaGaaeqabaqabeGadaaakeaacuWG4baEgaqeaaaa@2E3D@ and y¯
 MathType@MTEF@5@5@+=feaafiart1ev1aaatCvAUfKttLearuWrP9MDH5MBPbIqV92AaeXatLxBI9gBaebbnrfifHhDYfgasaacH8akY=wiFfYdH8Gipec8Eeeu0xXdbba9frFj0=OqFfea0dXdd9vqai=hGuQ8kuc9pgc9s8qqaq=dirpe0xb9q8qiLsFr0=vr0=vr0dc8meaabaqaciaacaGaaeqabaqabeGadaaakeaacuWG5bqEgaqeaaaa@2E3F@ are their corresponding means. Here *N *is the total residue number in a protein.

In order to compare the prediction performance with existing methods, we also used the same measure *DevA*_*p *_proposed by Kinjo *et al*. [11,12] to calculate the RMS error between the predicted and observed RWCO values

DevAp=∑i=1N(xi−yi)2∑i=1N(xi−x¯)2.     (10)
 MathType@MTEF@5@5@+=feaafiart1ev1aaatCvAUfKttLearuWrP9MDH5MBPbIqV92AaeXatLxBI9gBaebbnrfifHhDYfgasaacH8akY=wiFfYdH8Gipec8Eeeu0xXdbba9frFj0=OqFfea0dXdd9vqai=hGuQ8kuc9pgc9s8qqaq=dirpe0xb9q8qiLsFr0=vr0=vr0dc8meaabaqaciaacaGaaeqabaqabeGadaaakeaacqWGebarcqWGLbqzcqWG2bGDcqWGbbqqdaWgaaWcbaGaemiCaahabeaakiabg2da9maalaaabaWaaOaaaeaadaaeWbqaamaabmGabaGaemiEaG3aaSbaaSqaaiabdMgaPbqabaGccqGHsislcqWG5bqEdaWgaaWcbaGaemyAaKgabeaaaOGaayjkaiaawMcaamaaCaaaleqabaGaeGOmaidaaaqaaiabdMgaPjabg2da9iabigdaXaqaaiabd6eaobqdcqGHris5aaWcbeaaaOqaamaakaaabaWaaabCaeaadaqadiqaaiabdIha4naaBaaaleaacqWGPbqAaeqaaOGaeyOeI0IafmiEaGNbaebaaiaawIcacaGLPaaadaahaaWcbeqaaiabikdaYaaaaeaacqWGPbqAcqGH9aqpcqaIXaqmaeaacqWGobGta0GaeyyeIuoaaSqabaaaaOGaeiOla4IaaCzcaiaaxMaadaqadiqaaiabigdaXiabicdaWaGaayjkaiaawMcaaaaa@5959@

The root mean square error (RMSE) is also given by

RMSE=1N∑i=1N(yi−xi)2.     (11)
 MathType@MTEF@5@5@+=feaafiart1ev1aaatCvAUfKttLearuWrP9MDH5MBPbIqV92AaeXatLxBI9gBaebbnrfifHhDYfgasaacH8akY=wiFfYdH8Gipec8Eeeu0xXdbba9frFj0=OqFfea0dXdd9vqai=hGuQ8kuc9pgc9s8qqaq=dirpe0xb9q8qiLsFr0=vr0=vr0dc8meaabaqaciaacaGaaeqabaqabeGadaaakeaacqWGsbGucqWGnbqtcqWGtbWucqWGfbqrcqGH9aqpdaGcaaqaamaalaaabaGaeGymaedabaGaemOta4eaamaaqahabaWaaeWaceaacqWG5bqEdaWgaaWcbaGaemyAaKgabeaakiabgkHiTiabdIha4naaBaaaleaacqWGPbqAaeqaaaGccaGLOaGaayzkaaWaaWbaaSqabeaacqaIYaGmaaaabaGaemyAaKMaeyypa0JaeGymaedabaGaemOta4eaniabggHiLdaaleqaaOGaeiOla4IaaCzcaiaaxMaadaqadiqaaiabigdaXiabigdaXaGaayjkaiaawMcaaaaa@4A7B@

We computed two kinds of RMSE values: one is based on the predicted and observed normalized RWCO values (denoted by RMSE_norm) and the other is based on the predicted and observed raw (absolute) RWCO values (denoted by RMSE_raw).

## Authors' contributions

JS conceived the study, designed the methodology, developed the computer programs and drafted the manuscript. KB coordinated the project, supervised the process and refined the manuscript. All authors read and approved the final manuscript.

## Supplementary Material

Additional file 1 The ASTRAL SCOP codes of 680 protein sequences used in this study. This file contains the ASTRAL protein chain names and their corresponding amino acid sequences. Click here for file

Additional file 2 This file contains the protein chain names, their detailed contact number and residue-wise contact order information with a radius cutoff of 12Å. The second and third columns are the residue name and original residue position in ATOM records, respectively. The third and fourth columns are the discrete and consecutive contact numbers, respectively. And the last column is the observed residue-wise contact order. Click here for file
